# Prevalence of Medication-Overuse Headache and Awareness of Medication Overuse Among the General Population in Western Saudi Arabia: A Cross-Sectional Study

**DOI:** 10.7759/cureus.110467

**Published:** 2026-06-08

**Authors:** Thekra Albeshri, Shahad Albarqi, Hussam Al Malki, Khadija Alnasif, Ghidaa A Alghamdi, Taghred Aljohani, Fai Alotaibi, Muhammed T Bakhsh, Amal Alkhotani

**Affiliations:** 1 College of Medicine, Umm Al-Qura University, Makkah, SAU; 2 College of Medicine, Taif University, Taif, SAU; 3 College of Medicine, Fakeeh College of Medical Sciences, Jeddah, SAU; 4 College of Medicine, Taibah University, Madinah, SAU; 5 Medicine and Surgery, Umm Al-Qura University, Makkah, SAU; 6 Medicine, Umm Al-Qura University, Makkah, SAU

**Keywords:** headache prevalence, knowledge, medication overuse headache (moh), public awareness, saudi arabia

## Abstract

Introduction

Medication-overuse headache (MOH) is a common but often underdiagnosed secondary headache disorder that develops in individuals with pre-existing headache disorders as a result of excessive use of acute headache medications. This study aims to assess the estimated prevalence and awareness of MOH among the general population in the Western region of Saudi Arabia and to explore factors associated with headache frequency and medication use.

Methods

An observational cross-sectional study was conducted from December 2024 to January 2025 among adults aged 19-60 years residing in Western Saudi Arabia. Data were collected using an online self-administered questionnaire distributed via social media platforms. The tool collected data on sociodemographic factors, headache patterns, medication use, and MOH awareness. A total of 518 responses were included in the final analysis.

Results

A total of 518 participants completed the questionnaire; 362 (69.9%) were female, with a mean age of 31.2 ± 12.9 years. Overall, 490 participants (94.6%) reported a history of headaches. The estimated prevalence of MOH was 2.4%, while awareness among participants was 20.8% (n = 109). A significant association was observed between age and headache prevalence (p = 0.004), with the highest prevalence of 97.1% (n = 165) among participants aged 20-29 years. In addition, a significant association was found between age and medication use (p = 0.049), with a progressive increase observed: medication use increased from 71.4% (n = 85) in participants under 20 years to 85.5% (n = 59) in those aged 40-49 years. These findings reflect the direction and practical impact of age-related differences.

Conclusion

The study found a lower estimated prevalence of MOH compared to previous national studies, while awareness remained low in Western Saudi Arabia. These findings highlight the importance of educational initiatives and improved headache management strategies to address the risks of medication overuse.

## Introduction

The worsening of an underlying headache caused by the regular overuse of acute headache medications is known as medication-overuse headache (MOH). MOH is a widely prevalent but frequently underdiagnosed condition that arises from the excessive use of medications intended for acute headache relief. Unlike an acute headache, MOH is a chronic condition caused by excessive medication use. The International Classification of Headache Disorders (ICHD-3) categorizes MOH as a secondary headache disorder that develops due to the repetitive use of analgesics, triptans, and other pharmaceutical treatments for primary headache conditions, such as migraines and tension-type headaches [1]. According to ICHD-3, MOH is defined as a headache occurring on 15 or more days per month in individuals with a pre-existing primary headache disorder, developing after more than three months of regular use of acute headache medications, with overuse thresholds of 15 or more days per month for simple analgesics and 10 or more days per month for triptans, opioids, or combination analgesics [1].

Medication use refers to the appropriate administration of medications, including dosage and duration, as recommended by medical guidelines [2]. In contrast, medication misuse, abuse, and overuse are related but different concepts. Misuse refers to the use of medications in a manner that differs from medical guidelines, while abuse involves the non-therapeutic use of drugs to achieve psychoactive outcomes. Overuse refers to the excessive use of medications beyond recommended dosages or durations [3]. These practices are particularly relevant in the context of MOH, which is highly associated with frequent use of acute headache medications.

Moreover, MOH represents a significant public health concern because it diminishes quality of life, imposes a substantial economic burden, and complicates the clinical management of individuals with chronic headaches who develop a dependency on symptomatic medications.

Further, MOH is recognized as one of the leading causes of chronic daily headaches, affecting approximately 1% to 2% of the global population [4]. Its prevalence varies among demographic groups and is high among individuals with preexisting primary headache disorders, especially migraine [5], and is most commonly observed in middle-aged individuals, particularly during the fourth and fifth decades of life [6]. Studies have consistently indicated that MOH occurs more frequently in females than in males, with reported female-to-male ratios ranging from 2:1 to 5:1 [7]. This gender disparity may be attributed to a combination of biological and genetic factors, including hormonal fluctuations, variations in neurological function, genetic predisposition, stress exposure, and the higher incidence of migraines among females [8].

The economic consequences of MOH are substantial, as affected individuals often require frequent medical consultations, which contribute to rising healthcare expenditures and lost productivity. Beyond financial implications, MOH significantly impairs daily functioning and mental health, further reducing overall well-being [9].

Several risk factors have been associated with the development of MOH, including medication frequency and type, education level, and coexisting psychiatric conditions such as anxiety and depression [10]. Excessive use of specific medications, particularly combination analgesics, opioids, and triptans, has been strongly linked to an increased risk of developing MOH [11].

Although extensive research on MOH has been conducted in Western populations, data on its prevalence in Saudi Arabia remain limited. Given the high occurrence of migraines and other primary headaches in the Middle Eastern region, MOH is likely to be an increasing concern in Saudi communities [12]. Moreover, cultural and healthcare system factors, including the widespread availability of over-the-counter analgesics, may contribute to an increased risk of MOH development in this region [13]. Raising awareness of MOH among healthcare professionals and the public is crucial for early diagnosis and prevention. Studies have suggested that insufficient awareness often results in delayed diagnoses and places additional strain on healthcare resources [14]. Although a previous study explored MOH prevalence and awareness in Makkah, comprehensive research across the broader Western region of Saudi Arabia remains lacking. Therefore, this study aims to estimate the prevalence of MOH, assess awareness among adults in Western Saudi Arabia, and explore factors associated with headache frequency and medication use.

## Materials and methods

Study design and participants

A cross-sectional study was conducted from December 2024 to January 2025 to assess the prevalence and awareness of MOH among the population in the Western region of Saudi Arabia. The study targeted residents of the Western region of Saudi Arabia aged 19 to 60 years, regardless of nationality or gender, who were literate and had active social media accounts. Participants were recruited through a non-probability convenience sampling approach using an online self-administered questionnaire distributed via Google Forms (Google LLC, Mountain View, CA, USA) across social media platforms. While this approach facilitated broad and rapid outreach, it may have introduced selection bias by favoring participants who are more active online and more educated.

The inclusion criteria included residents of the Western region of Saudi Arabia aged 19 to 60 years who could read and understand the questionnaire and had access to social media platforms. Individuals residing outside the Western region, or those younger than 19 or older than 60, were excluded. The questionnaire settings required all items to be completed before submission; thus, no incomplete responses were included in the analysis. No duplicate responses were detected, as Google Forms was configured to allow only one response per verified Google account. Information regarding participants' pre-existing comorbidities was not collected as part of the study questionnaire and was considered a study limitation.

Ethical considerations

Ethical approval was obtained from the Biomedical Research Ethics Committee at Umm Al-Qura University (approval number: HAPO-02-K-012-2024-12-2411).

Sample size

The sample size was calculated using OpenEpi (version 3.0; Dean AG, Sullivan KM, Soe MM. OpenEpi: Open Source Epidemiologic Statistics for Public Health), assuming a 95% confidence level, a margin of error of 5%, and an anticipated population proportion of 50%, resulting in a minimum required sample size of 385 participants. The calculated sample size was increased by approximately 20% to account for possible non-response or incomplete data, resulting in a final target sample of 471 individuals. A total of 518 participants were surveyed, and all responses were included in the final analysis, as no incomplete submissions were identified.

Study tool and scoring system

The questionnaire was adapted from previously published Saudi studies [14-16]. Ten participants in a validation study assessed the questionnaire’s clarity and accuracy; those participants were not included in the final study sample. Following validation, the questionnaire was administered to the study participants.

The questionnaire included the following three sections: (1) Sociodemographic data: This section included information on nationality, gender, age, residential city, and education level. (2) Headache assessment: This section consisted of two components: (a) whether the participant had ever experienced a headache, and (b) the number of days they experienced headaches within the previous 30 days. (3) Medication use: This section consisted of four components: (a) the type of medications used to relieve headaches, (b) the number of days headache medications were used within the previous 30 days, (c) the usual duration of medication use, and (d) participants’ awareness of MOH, assessed by a question regarding the side effects of headache medications (see Appendices).

MOH classification was based on a scoring system derived from participants’ responses to four questions. The maximum possible score was 11, and a total score of 8 or higher was used to identify probable MOH in this survey (Table 1). While the scoring system was not designed to directly measure ICHD-3 diagnostic criteria, it was adapted from previously published survey instruments used in comparable Saudi populations [15,16] and should be interpreted as a survey-based estimated case definition rather than a formal clinical diagnosis.

**Table 1:** Scoring system used for MOH classification MOH: medication-overuse headache

Question	No points	1 point	2 points	3 points	4 points
For how many days have you experienced headaches within the last 30 days?	Did not experience headaches	1-2 days	3-7 days	8-14 days	15 days or more
Do you use any medication to relieve your headache?	Does not use headache medications	Uses headache medications	-	-	-
For how many days have you used these medications to relieve your headache within the last 30 days?	Did not use headache medications within the last 30 days	1-9 days	10-14 days	15-30 days	-
For how long do you usually use these medications to relieve your headache?	Intermittently	Less than 1 month	1-3 months	More than 3 months	-

The scoring system was based on four questions. The first question asked, “For how many days have you experienced headaches within the last 30 days?” Responses were scored as follows: “Did not experience headaches within the last 30 days” (0 points), “1-2 days” (1 point), “3-7 days” (2 points), “8-14 days” (3 points), and “15 days or more” (4 points).

The second question, “Which of the following medications do you use to relieve your headache?”, was assigned one point for participants who reported using headache medications, including paracetamol, nonsteroidal anti-inflammatory drugs (NSAIDs), Solpadeine, or sumatriptan.

The third question asked, “For how many days have you used these medications to relieve your headache within the last 30 days?” Responses were scored as follows: “Did not use headache medications within the last 30 days” (0 points), “1-9 days” (1 point), “10-14 days” (2 points), and “15-30 days” (3 points).

The fourth question asked, “For how long do you usually use these medications to relieve your headache?” Responses were scored as follows: “Intermittently” (0 points), “less than 1 month” (1 point), “1-3 months” (2 points), and “more than 3 months” (3 points).

Additionally, the questionnaire assessed MOH awareness by asking about possible side effects of these medications using a multiple-choice checkbox question (one option was chronic headache). Those who chose chronic headache as a potential side effect were considered aware of MOH.

Data analysis

Data analysis was conducted using IBM SPSS Statistics for Windows, Version 27 (Released 2019; IBM Corp., Armonk, New York, United States). Descriptive statistics were employed to summarize the sociodemographic characteristics, headache experience and frequency, awareness of side effects, and medication use patterns among participants. Frequencies and percentages were calculated for categorical variables, and the means and standard deviations were computed for continuous variables. Various statistical tests were applied to explore factors associated with headache prevalence and medication use. The Pearson chi-square test was employed to assess the association between categorical variables (e.g., city, gender, and nationality) and headache prevalence and medication use. Exact probability tests were employed when necessary for small-frequency distributions. Additionally, the distribution of medication use according to headache frequency was examined. The relationship between the number of days medications were used and the frequency of headache-relief medication use was evaluated using the exact probability test (p < 0.05 is considered statistically significant).

## Results

Table 2 provides a detailed overview of the sociodemographic characteristics of the study participants in the Western region of Saudi Arabia. Most participants were from Jeddah (34.7%, n = 180) and Makkah (25.1%, n = 130), with a smaller representation from Taif (17.8%, n = 92), other cities (13.3%, n = 69), and Madinah (9.1%, n = 47). The mean age of participants was 31.2 years, with a standard deviation of 12.9 years. A significant portion of the participants were aged 20 to 29 years (32.8%, n = 170) and under 20 years (26.8%, n = 139), followed by those 40 to 49 years (15.8%, n = 82), 50 years and older (13.5%, n = 70), and 30 to 39 years (11.0%, n = 57). Female participants constituted a higher proportion of the sample (69.9%, n = 362) than male participants (30.1%, n = 156). Regarding nationality, the vast majority were Saudi nationals (92.7%, n = 480), with a small percentage of non-Saudi participants (7.3%, n = 38). Regarding educational level, a considerable proportion of participants held a bachelor’s degree (62.0%, n = 321), followed by those with secondary education (30.1%, n = 156), below secondary education (5.8%, n = 30), and post-graduate degrees (2.1%, n = 11).

**Table 2:** Socio-demographic characteristics of study participants in the Western region, Saudi Arabia (n = 518)

Socio-demographics	Number (%)
City	
Jeddah	180 (34.7)
Makkah	130 (25.1)
Taif	92 (17.8)
Other	69 (13.3)
Madinah	47 (9.1)
Age in years	
<20	139 (26.8)
20-29	170 (32.8)
30-39	57 (11.0)
40-49	82 (15.8)
50+	70 (13.5)
Mean ± SD = 31.2 ± 12.9	
Gender	
Male	156 (30.1)
Female	362 (69.9)
Nationality	
Saudi	480 (92.7)
Non-Saudi	38 (7.3)
Educational level	
Below secondary	30 (5.8)
Secondary education	156 (30.1)
Bachelor degree	321 (62.0)
Post-graduate degree	11 (2.1)

**Figure 1:**
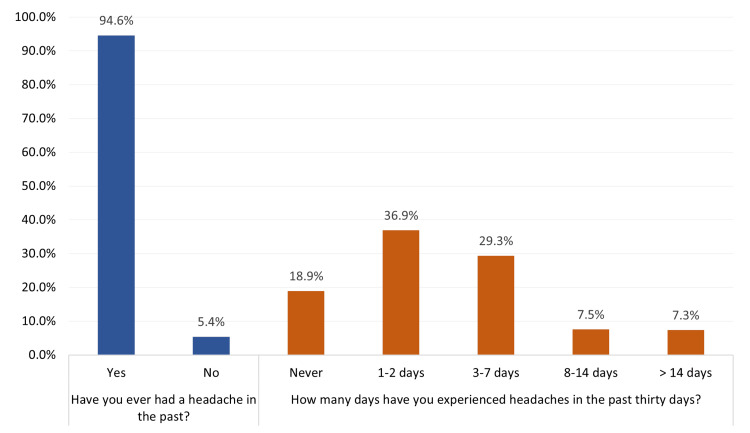
Headache experience and frequency among participants in Western region, Saudi Arabia

Figure 1 presents the headache experience and frequency among participants for the past 30 days. A significant majority of participants (94.6%, n = 490) reported experiencing a headache at some point, whereas a small proportion (5.4%, n = 28) reported never having had a headache. Regarding headache frequency in the past 30 days, 36.9% of participants (n = 191) experienced headaches on one to two days, followed by 29.3% (n = 152) who experienced headaches on three to seven days, and 18.9% (n = 98) who did not experience any headaches. Additionally, 7.5% of participants (n = 39) reported experiencing headaches for 8 to 14 days, and 7.3% (n = 38) experienced headaches for more than 14 days.

Table 3 lists the types of headache medications used by participants and their frequency of use. The most commonly used medication was paracetamol, taken by 69.1% of the participants (n = 291), followed by those who did not use any medication (23.0%, n = 97). Other medications used include Solpadeine (5.2%, n = 22), NSAIDs (2.1%, n = 9), and sumatriptan (0.5%, n = 2). Regarding the frequency of medication use in the past 30 days, most participants (89.5%, n = 290) used medications for one to nine days. Smaller percentages of respondents used medications for 10 to 14 days (5.6%, n = 18) and 15 to 30 days (4.9%, n = 16). When considering how often these drugs are used, most participants (75.3%, n = 244) reported using them intermittently. Others used them for less than a month (11.7%, n = 38), one to three months (8.6%, n = 28), and more than three months (4.3%, n = 14).

**Table 3:** Medications used to relieve headaches and their frequency of use among participants (n = 421) NSAIDs: nonsteroidal anti-inflammatory drugs

Medications	Number (%)
Medications used to relieve headaches	
I don't use any medications	97 (23.00)
Paracetamol	291 (69.10)
Solpadeen	22 (5.20)
NSAIDs	9 (2.10)
Sumatriptan	2 (0.50)
How many days have you used previous medications to relieve headaches in the last 30 days?	
1-9 days	290 (89.50)
10-14 days	18 (5.60)
15-30 days	16 (4.90)
How often are the previous drugs used to relieve headaches?	
Intermittently	244 (75.30)
Less than a month	38 (11.70)
1-3 months	28 (8.60)
More than 3 months	14 (4.30)

Table 4 shows that, using the applied scoring system, the estimated prevalence of MOH was 2.4% among the 490 participants who reported experiencing headaches.

**Table 4:** The results of the scoring system

Score	Number	%
0	25	5.10
1	66	13.47
2	62	12.65
3	94	19.18
4	117	23.88
5	58	11.84
6	34	6.94
7	22	4.49
8	6	1.22
9	3	0.61
10	2	0.41
11	1	0.20
Total	490	100.00

**Figure 2:**
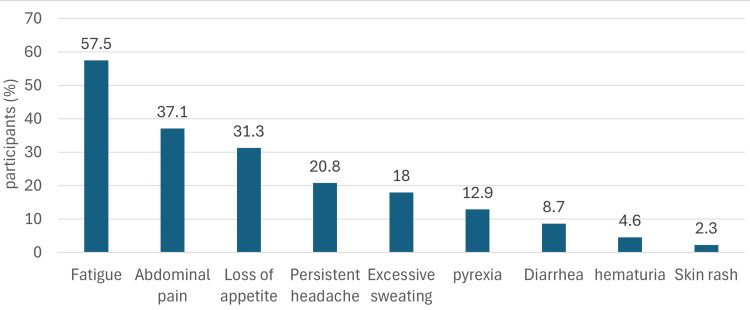
Participants’ knowledge of possible side effects associated with the headache medications included in the study in the Western region, Saudi Arabia (n = 518)

Figure 2 presents participants’ knowledge of the potential side effects (defined as unintended adverse effects of medications) associated with headache medications. The most commonly known side effect was fatigue, reported by 57.5% (n = 300) of the participants. Abdominal pain was the second most common side effect, reported by 37.1% (n = 192) of participants, followed by loss of appetite (31.3%, n = 162) and persistent headache (20.8%, n = 109). Other side effects included excessive sweating (18.0%, n = 94), pyrexia (12.9%, n = 68), diarrhea (8.7%, n = 45), hematuria (4.6%, n = 25), and skin rash (2.3%, n = 13).

**Figure 3:**
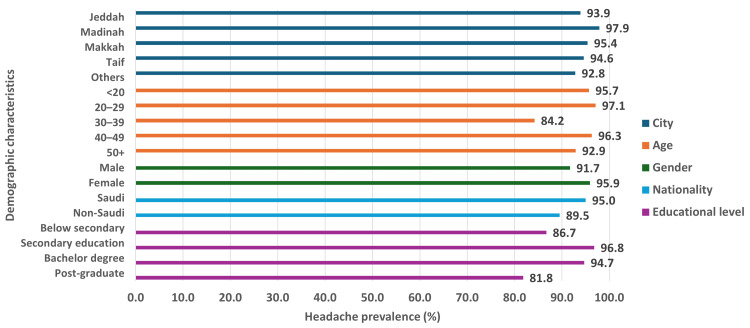
Headache prevalence according to demographic characteristics among participants in the Western region, Saudi Arabia

Figure 3 presents the distribution of headache prevalence according to demographic characteristics among study participants in the Western region of Saudi Arabia, providing important context for identifying patterns that may contribute to MOH. The prevalence of headaches is relatively consistent across different cities, with Madinah having the highest percentage of participants reporting headaches (97.9%, n = 46), followed by Makkah (95.4%, n = 124), Taif (94.6%, n = 87), Jeddah (93.9%, n = 169), and other cities (92.8%, n = 64) (p = 0.766; Cramér’s V = 0.06). A significant association was observed between age and headache prevalence (p = 0.004; Cramér's V = 0.17). Participants aged 20-29 years reported the highest prevalence of headaches (97.1%, n = 165). A higher percentage of female participants (95.9%, n = 347) reported headaches than male participants (91.7%, n = 143) (p = 0.048; Cramér’s V = 0.09). The difference in headache prevalence between Saudi and non-Saudi participants was not statistically significant (p = 0.147; Cramér's V = 0.06). A significant association was observed between educational level and headache prevalence (p = 0.034; Cramér’s V = 0.13).

**Figure 4:**
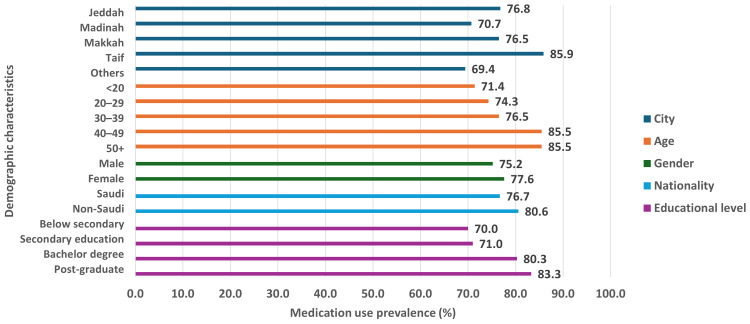
Headache medication use according to demographic characteristics among participants in the Western region, Saudi Arabia

Figure 4 presents the distribution of headache medication use according to demographic characteristics among study participants in the Western region of Saudi Arabia, which is a key contributor to the development of MOH. Medication use varied by city (p = 0.198; Cramér’s V = 0.12). Age showed a significant association (p = 0.049; Cramér's V = 0.14), with the highest use among participants aged 40+ years. No significant association was found between gender and medication use (p = 0.598; Cramér's V = 0.02) or nationality (p = 0.613; Cramér's V = 0.02). Educational level was also not significantly associated (p = 0.173; Cramér’s V = 0.10).

Table 5 evaluated medication use relative to headache frequency. Although medication use increased with headache frequency (e.g., 73.3% (n = 140) for 1-2 days vs. 84.2% (n = 32) for >14 days), the association was not statistically significant (p = 0.146; Cramér's V = 0.12). However, the number of days medications were used was significantly associated with headache frequency (p = 0.001; Cramér’s V = 0.24), indicating a moderate effect size. No significant association was observed in the frequency of medication use (p = 0.456; Cramér’s V = 0.14).

**Table 5:** The distribution of medication use by frequency of headache among study participants, Western region, Saudi Arabia P: Exact probability test; * P < 0.05 (significant)

Medication use	Frequency of headache								p-value
	1-2 days		3-7 days		8-14 days		>14 days		
	Number	%	Number	%	Number	%	Number	%	
Medication use to relieve headaches									
Yes	140	73.3	120	78.9	32	82.1	32	84.2	0.146
No	51	26.7	32	21.1	7	17.9	6	15.8	
How many days have you used previous medications to relieve headaches in the last 30 days?									
1-9 days	134	95.7	109	90.8	26	81.3	21	65.6	0.001*
10-14 days	2	1.4	7	5.8	5	15.6	4	12.5	
15-30 days	4	2.9	4	3.3	1	3.1	7	21.9	
How often are the previous drugs used to relieve headaches?									
Intermittently	109	77.9	89	74.2	22	68.8	24	75.0	0.456
Less than a month	13	9.3	16	13.3	7	21.9	2	6.3	
1-3 months	13	9.3	9	7.5	3	9.4	3	9.4	
More than 3 months	5	3.6	6	5.0	0	0.0	3	9.4	

## Discussion

MOH is a global health concern and a form of secondary chronic daily headache that develops in individuals with underlying primary headache disorders as a result of excessive use of acute headache medications. It is considered the third most common headache disorder worldwide, following tension-type headache and migraine [17]. The prevalence of MOH has been reported to be approximately 1-2%, although it may reach up to 7% in certain populations, with higher rates observed among women and individuals from lower socioeconomic backgrounds [7]. Understanding its prevalence and public awareness may help inform efforts to reduce its impact on patients’ quality of life.

In the present study, the estimated prevalence of MOH was 2.4%. This finding is lower than that reported in previous Saudi studies conducted in Qassim province (4.0%) and Makkah city (4.5%) [15,16]. Nevertheless, it is comparable to international estimates, including studies from Japan (2.3%), Denmark (2.0%), Spain (1.4%), and Lithuania (3.2%) [18-21]. The lower estimated prevalence may partly reflect characteristics of the study sample, including the relatively high educational level of participants: 62% (n = 321) held a bachelor’s degree.

Despite the lower estimated prevalence of MOH observed in this study, awareness remains a major concern, with only 20.8% (n = 109) of participants recognizing chronic headache as a potential consequence of medication overuse. This level is comparable to previous Saudi studies reporting awareness levels of 18.0%-18.7% [15,16]. Overall awareness may be overestimated, as the sample included a considerable proportion of well-educated participants. These findings suggest that public health efforts to improve awareness of the risks of excessive medication use may be beneficial.

In the present study, a high prevalence of headache (94.6%, n = 490) was observed, and this finding should be interpreted with caution. It may be partially attributable to the social media recruitment strategy, which could have attracted individuals more interested in headache-related topics or with personal experience with headaches, thereby increasing their likelihood of participating in the survey. Furthermore, headache was defined broadly in the questionnaire as any past history of headache rather than a clinically confirmed diagnosis, potentially leading to an overestimation of headache prevalence in the general population.

MOH may create a cycle in which more frequent headache episodes can lead to increased analgesic use, which may, in turn, contribute to further headache recurrence. In this study, medication use tended to increase with headache frequency, although this association was not statistically significant. However, the number of days medications were used was significantly associated with headache frequency, whereas the usual duration of medication use was not. This pattern may reflect differences in headache severity or other unmeasured variables. Previous studies have suggested that withdrawal may be more difficult among patients with frequent headaches or those overusing opioids or combination analgesics [22]. In one study, starting prophylactic therapy at the time of withdrawal was associated with a 60% reduction in headache frequency over six months [23]. These findings suggest that heightened awareness and appropriate management may play a role in reducing excessive medication use and its associated consequences.

A statistically significant association was observed between age and headache prevalence (p = 0.004). The highest prevalence was reported by participants aged 20 to 29 years (97.1%, n = 165), followed by those aged under 20 years (95.7%, n = 133), those aged 40 to 49 years (96.3%, n = 79), and those aged 50 years and older (92.9%, n = 65). The lowest prevalence was observed in the 30-39 age group (84.2%, n = 48). These findings are consistent with the global literature, indicating that adolescents and young adults experience the highest burden of headache disorders. For instance, a systematic review by Stovner et al. (2018) demonstrated that individuals aged 15 to 39 years are disproportionately affected by headache disorders, supporting the patterns observed in the cohort [24]. The comparatively lower prevalence among individuals aged 30 to 39 years, compared with those aged 40 to 49 years, may reflect unmeasured lifestyle or occupational factors, but this should be interpreted cautiously. Further research is needed to determine whether this discrepancy is specific to the population or reflects a broader epidemiological trend.

Additionally, a statistically significant relationship was observed between age and medication use for headache management (p = 0.049). The highest rates of medication use were reported among participants aged 40 to 49 years (85.5%, n = 59) and those aged 50 years and older (85.5%, n = 47), followed by those aged 30 to 39 years (76.5%, n = 26), those aged 20 to 29 years (74.3%, n = 107), and those aged under 20 years (71.4%, n = 85). This pattern may reflect greater healthcare utilization, a higher burden of comorbid conditions, or differences in treatment-seeking behavior among older adults, although these factors were not directly assessed in this study. These findings suggest that age-tailored headache management strategies integrating pharmacological and nonpharmacological approaches may help mitigate the risk of medication overuse [24].

This study has several limitations. The data were collected using a self-reported questionnaire, which could lead to recall bias and misinterpretation of the questions. In addition, the use of an online non-probability convenience sampling approach via social media platforms may have introduced selection bias by favoring individuals who were more active online and had higher levels of education. As most participants held a bachelor’s degree, the study sample may not fully represent the broader population, potentially leading to an overestimation of awareness levels and limiting the generalizability of the findings beyond populations accessible through social media platforms in the Western region of Saudi Arabia. Furthermore, this study did not investigate crucial factors that may substantially influence medication use, such as socioeconomic status, access to healthcare facilities, cultural beliefs about medication use, and pre-existing neurological disorders or chronic conditions known to significantly affect headache patterns or medication use. In addition, reliance on a questionnaire-based scoring approach rather than a full clinical assessment may have affected the accuracy of the estimated prevalence and classification of MOH. Additionally, the analyses were primarily descriptive and univariate, which limited the ability to explore independent factors associated with medication use patterns, awareness, or MOH classification. Therefore, further studies incorporating socioeconomic data and more comprehensive study designs are necessary to better identify the factors contributing to MOH and support the development of focused and effective preventive measures.

## Conclusions

The study found a lower estimated prevalence of MOH than previous national studies and a prevalence closer to international estimates. Awareness of the condition remained low among adults in Western Saudi Arabia. A considerable proportion of participants relied on over-the-counter medications, particularly paracetamol, despite limited awareness of their potential risks. These findings underscore the need for targeted educational initiatives, improved headache management, and greater involvement of healthcare professionals to reduce the burden of medication overuse and promote safer medication practices.

## Appendices

The Questionnaire

1. Demographics

(1) Age (in years)

(2) Gender:

A. Male

B. Female

(3) Nationality:

A. Saudi

B. Non-Saudi

(4) Residential City:

A. Makkah

B. Jeddah

C. Madinah

D. Taif

E. Others

(5) What is your educational level?

A. Less than secondary school

B. Secondary school

C. Bachelor’s degree

D. Master’s degree

E. Ph.D.

2. Headache Assessment

(6) Have you ever experienced a headache in the past?

A. Yes

B. No

(7) For how many days have you experienced headaches within the last 30 days?

A. Did not experience any headaches.

B. 1-2 days

C. 3-7 days

D. 8-14 days

E. 15 days or more

3. Medication Use

(8) Which of the following medications do you use to relieve your headache?

A. I don’t use medications for headache

B. Paracetamol (Panadol, Fevadol, Adol, Panadrex, or Acitam)

C. NSAIDs (aspirin, ibuprofen, or diclofenac)

D. Solpadeine

E. Sumatriptan

(9) For how many days have you used these medications to relieve your headache within the last 30 days?

A. Did not use headache medications within the last 30 days.

B. 1-9 days

C. 10-14 days

D. 15-30 days

(10) For how long do you usually use these medications to relieve your headache?

A. Intermittently

B. Less than one month

C. 1-3 months

D. More than three months

(11) Please select all the headache medication side effects that you are aware of (more than one answer can be selected):

• Diarrhea

• Increased sweating

• Loss of appetite

• Stomach cramps

• Skin rash

• Chronic headache

• Fever

• Red-colored urine

• Fatigue or exhaustion 

## References

[1] (2018). Headache Classification Committee of the International Headache Society (IHS) The International Classification of Headache Disorders, 3rd edition. Cephalalgia.

[2] World Health Organization (1987). The Rational Use of Drugs: Report of the Conference of Experts, Nairobi, 25-29 November 1985. The rational use of drugs: report of the conference of experts, Nairobi, 25-29 November 1985.

[3] Smith SM, Dart RC, Katz NP (2013). Classification and definition of misuse, abuse, and related events in clinical trials: ACTTION systematic review and recommendations. Pain.

[4] Diener HC, Holle D, Solbach K, Gaul C (2016). Medication-overuse headache: risk factors, pathophysiology and management. Nat Rev Neurol.

[5] Hagen K, Jensen R, Bøe MG, Stovner LJ (2010). Medication overuse headache: a critical review of end points in recent follow-up studies. J Headache Pain.

[6] Kulkarni GB, Mathew T, Mailankody P (2021). Medication overuse headache. Neurol India.

[7] Westergaard ML, Munksgaard SB, Bendtsen L, Jensen RH (2016). Medication-overuse headache: a perspective review. Ther Adv Drug Saf.

[8] Allais G, Chiarle G, Sinigaglia S, Airola G, Schiapparelli P, Benedetto C (2020). Gender-related differences in migraine. Neurol Sci.

[9] Benz T, Nüssle A, Lehmann S (2017). Health and quality of life in patients with medication overuse headache syndrome after standardized inpatient rehabilitation: a cross-sectional pilot study. Medicine (Baltimore).

[10] Fischer MA, Jan A (2025). Medication-overuse headache. StatPearls [Internet].

[11] González-Oria C, Belvís R, Cuadrado ML (2021). Document of revision and updating of medication overuse headache (MOH). Neurologia (Engl Ed).

[12] Al Jumah M, Al Khathaami AM, Kojan S, Hussain M, Thomas H, Steiner TJ (2020). The prevalence of primary headache disorders in Saudi Arabia: a cross-sectional population-based study. J Headache Pain.

[13] Mannasaheb BA, Alajlan SA, Alshahrani JA (2022). Prevalence, predictors and point of view toward self-medication among residents of Riyadh, Saudi Arabia: a cross-sectional study. Front Public Health.

[14] Alharbi B, Anjum I, Altasan A, Alhussain M, Alshammasi H, Alfunaysan A, Masudi E (2021). Exploration of Saudi’s general population’s awareness about paracetamol (acetaminophen) overuse headache: a cross-sectional inquiry in Riyadh, Saudi Arabia. Int J Med Dev Ctries.

[15] Almuqairsha SA, Aldekhail MI, Aldekhail AI (2022). The prevalence and level of awareness of medication overuse headache in Qassim province, Saudi Arabia: a cross-sectional study. Cureus.

[16] Alharbi AS, Alharbi OF, Qutub FL (2023). Assessment of the prevalence and level of awareness of medication overuse headache among the general population in Makkah City, Saudi Arabia. Cureus.

[17] Jonsson P, Hedenrud T, Linde M (2011). Epidemiology of medication overuse headache in the general Swedish population. Cephalalgia.

[18] Katsuki M, Yamagishi C, Matsumori Y (2022). Questionnaire-based survey on the prevalence of medication-overuse headache in Japanese one city-Itoigawa study. Neurol Sci.

[19] Westergaard ML, Lau CJ, Allesøe K, Gjendal ST, Jensen RH (2020). Monitoring chronic headache and medication-overuse headache prevalence in Denmark. Cephalalgia.

[20] Colás R, Muñoz P, Temprano R, Gómez C, Pascual J (2004). Chronic daily headache with analgesic overuse: epidemiology and impact on quality of life. Neurology.

[21] Rastenytė D, Mickevičienė D, Stovner LJ, Thomas H, Andrée C, Steiner TJ (2017). Prevalence and burden of headache disorders in Lithuania and their public-health and policy implications: a population-based study within the Eurolight Project. J Headache Pain.

[22] Deighton AM, Harris LA, Johnston K, Hogan S, Quaranta LA, L'Italien G, Coric V (2021). The burden of medication overuse headache and patterns of switching and discontinuation among triptan users: a systematic literature review. BMC Neurol.

[23] Bendtsen L, Munksgaard S, Tassorelli C (2014). Disability, anxiety and depression associated with medication-overuse headache can be considerably reduced by detoxification and prophylactic treatment. Results from a multicentre, multinational study (COMOESTAS project). Cephalalgia.

[24] Stovner LJ, Nichols E, Steiner TJ (2018). Global, regional, and national burden of migraine and tension-type headache, 1990-2016: a systematic analysis for the Global Burden of Disease Study 2016. Lancet Neurol.

